# The complete chloroplast genome sequence of *Aphanamixis polystachya*

**DOI:** 10.1080/23802359.2019.1688108

**Published:** 2019-11-12

**Authors:** Xiaolong Yuan, Yi Wang

**Affiliations:** Laboratory of Forest Plant Cultivation and Utilization, Yunnan Academy of Forestry, Kunming, People’s Republic of China;

**Keywords:** *Aphanamixis polystachya*, chloroplast, Illumina sequencing, phylogenetic analysis

## Abstract

The first complete chloroplast genome (cpDNA) sequence of *Aphanamixis polystachya* was determined from Illumina HiSeq pair-end sequencing data in this study. The cpDNA is 160,236 bp in length, contains a large single-copy region (LSC) of 87,484 bp and a small single-copy region (SSC) of 18,670 bp, which were separated by a pair of inverted repeats (IR) regions of 27,040 bp. The genome contains 130 genes, including 85 protein-coding genes, 8 ribosomal RNA genes, and 37 transfer RNA genes. The overall GC content of the whole genome is 37.6%, and the corresponding values of the LSC, SSC, and IR regions are 35.6%, 31.8%, and 42.8%, respectively. Further phylogenomic analysis showed that *A. polystachya* and *Cipadessa cinerascens* clustered in a clade in family Meliaceae.

*Aphanamixis polystachya* (Wall.) R. N. Parker belongs to Meliaceae family, is an evergreen broadleaf tree, and is a widespread found in south China, India, Pakistan, Bangladesh, Sri Lanka, and Malaysia (Palash et al. 2015). The extracts of *A. polystachya* have several bioactive such as antibacterial, antineoplastic, analgesic, and cytotoxic activities (Gude et al. 2019). Specific limonoids in this plant could control anticancer activity of various human cancer cell lines (Mulholland and Naidoo 1999; Shaikh et al. 2012). High economical and pharmaceutical potential of the abundant secondary metabolic compounds from *A. polystachya* have brought more attention into light for close supervision and scientific research (Shadid et al. 2016). However, there have been no genomic studies about *A. polystachya*.

Herein, we reported and characterized the complete *A. polystachya* plastid genome (MN106249). One *P. tomentosa* individual (specimen number: 201807017) was collected from Puwen, Yunnan Province of China (22°24′13′′N, 101°7′17′′E). The specimen is stored at Yunnan Academy of Forestry Herbarium, Kunming, China and the accession number is YAFH0012746. DNA was extracted from its fresh leaves using DNA Plantzol Reagent (Invitrogen, Carlsbad, CA).

Paired-end reads were sequenced by using Illumina HiSeq system (Illumina, San Diego, CA). In total, about 25.7 million high-quality clean reads were generated with adaptors trimmed. Aligning, assembly, and annotation were conducted by CLC de novo assembler (CLC Bio, Aarhus, Denmark), BLAST, GeSeq (Tillich et al. 2017), and GENEIOUS version 11.0.5 (Biomatters Ltd, Auckland, New Zealand). To confirm the phylogenetic position of *A. polystachya*, other nine species of family *Meliaceae* from NCBI were aligned using MAFFT version 7 (Katoh and Standley 2013). The Auto algorithm in the MAFFT alignment software was used to align the eight complete genome sequences and the G-INS-i algorithm was used to align the partial complex sequecnces and maximum likelihood (ML) bootstrap analysis was conducted using RAxML (Stamatakis 2006); bootstrap probability values were calculated from 1000 replicates. *Ailanthus altissima* (MG799542) and *Azadirachta indica* (KF986530) were served as the out-group.

The complete *A. polystachya* plastid genome is a circular DNA molecule with the length of 160,236 bp, contains a large single-copy region (LSC) of 87,484 bp and a small single-copy region (SSC) of 18,670 bp, which were separated by a pair of inverted repeats (IR) regions of 27,040 bp. The overall GC content of the whole genome is 37.6%, and the corresponding values of the LSC, SSC, and IR regions are 35.6%, 31.8%, and 42.8%, respectively. The plastid genome contained 130 genes, including 85 protein-coding genes, 8 ribosomal RNA genes, and 37 transfer RNA genes. Phylogenetic analysis showed that *A. polystachya* and *Cipadessa cinerascens* clustered in a unique clade in family *Meliaceae* (Figure 1). The determination of the complete plastid genome sequences provided new molecular data to illuminate the *Meliaceae* evolution.

**Figure 1.**
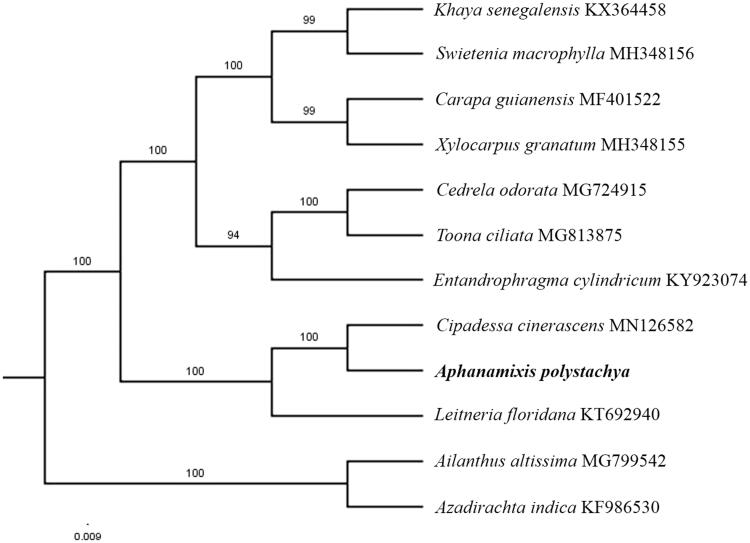
The maximum-likelihood tree based on the 10 chloroplast genomes of *Meliaceae*. The bootstrap value based on 1000 replicates is shown on each node.
